# Renal disease and diabetes increase the risk of failed outpatient management of cellulitic hand infections: a retrospective cohort study

**DOI:** 10.1186/s13018-023-03911-5

**Published:** 2023-06-10

**Authors:** Michael Allen, Joshua Gluck, Emily Benson

**Affiliations:** 1 Community Memorial Health System, 147 Brent St, Ventura, CA 93003 USA; 2 St. John’s Regional Medical Center, 1600 N Rose Ave, Oxnard, CA 93030 USA; 3 Ventura County Medical Center, 300 Hillmont Ave, Ventura, CA 93003 USA

**Keywords:** Infection, Hand, Upper extremity, Cellulitis, Antibiotics, Diabetes, Renal failure

## Abstract

**Background:**

Hand infections are heterogeneous, and some may undergo successful outpatient management. There are no strict guidelines for determining which patients will likely require inpatient admission for successful treatment, and many patients succeed with outpatient therapy. We sought to determine risk factors for failed outpatient management of cellulitic hand infections.

**Methods:**

We performed a retrospective review of patients who presented to the Emergency Department (ED) for hand cellulitic infections over five years, from 2014 to 2019. Vital signs, lab markers, Charlson Comorbidity Index (CCI), Elixhauser Comorbidity Measure (ECM), and antibiotic use were investigated. Discharge from the ED without subsequent admission was considered an outpatient success, while admission within 30 days of the prior visit was considered a failure. Continuous variables were compared with Welch's t test, and categorical data with Fisher's exact tests. Multivariable logistic regression was performed on comorbidities. Multiple testing adjustment was performed on p-values to generate q-values.

**Results:**

Outpatient management was attempted for 1,193 patients. 31 (2.6%) infections failed treatment, and 1,162 (97.4%) infections succeeded. Attempted outpatient treatment was 97.4% successful. Multivariable analysis demonstrated higher odds of failure with renal failure according to both CCI (OR 10.2, *p* < 0.001, *q* = 0.002) and ECM (OR 12.63, *p* = 0.003, *q* = 0.01) and with diabetes with complications according to the CCI (OR 18.29, *p* = 0.021, *q* = 0.032).

**Conclusions:**

Outpatient treatment failure was higher in patients with renal failure and complicated diabetes. These patients require a high index of suspicion for outpatient failure. These comorbidities should influence consideration for inpatient therapy though most patients can undergo successful treatment as outpatients.

**Level of evidence:**

Level III.

**Supplementary Information:**

The online version contains supplementary material available at 10.1186/s13018-023-03911-5.

## Background

Hand infections can have variable presentations and outcomes. Although commonplace, the literature lacks strong recommendations regarding the initial triage and definitive management of infections that are not surgical emergencies. Individual patient factors also further confuse the treatment picture. A superficial infection in an otherwise healthy host could have a different natural course than that in a patient with multiple comorbidities.

The incidence of finger infections is increasing, and the treatment frequently requires close integration of Emergency Department (ED), Infectious Disease, and Orthopaedic surgery specialties for effective diagnosis and management [1]. Prompt recognition and appropriate treatment are required to minimize morbidity and expedite recovery [2]. Upon presenting to the ED for initial triage, a thorough medical history, clinical examination, and the appropriate laboratory testing are paramount to the proper diagnosis and disposition. The clinical acumen of the ED provider is the first stage in deciding the appropriate treatment pathway. Many straightforward infections can be clinically diagnosed and receive appropriate therapy without further testing. The experienced ED provider may also determine which patients require Orthopaedic consultation for potential surgical management.

An outpatient management strategy is appropriate for many infections and minimizes excess healthcare expenditures. The primary purpose of this study was to determine patient-specific risk factors which portend a poor response to outpatient management of cellulitic hand infections. We hypothesized an increased risk of failure with increasing comorbidities.

## Methods

We performed a retrospective review of patients presenting to the ED at three major hospital systems providing care in our county from January 2014 through April 2019. These included one county Level II trauma center with a second satellite hospital and two Level III community systems, each with two hospitals. These three hospital systems provide care in our suburban region, which is geographically isolated, with the closest Level I center 33 miles away and the closest community hospital 18 miles away. Including these hospital networks allowed us to identify any patient seen for the same infection at multiple hospitals in our area. Each institution granted IRB approval.

All ED encounters containing a chosen upper extremity infection International Classification of Diseases (ICD) code for patients 18 years or older were extracted. The ICD-9-CM and ICD-10 codes covered cellulitis, lymphangitis, and infected abrasions of the hand and fingers (Additional file 1). We excluded charts if there were codes for bites, abscess, felon, septic arthritis, flexor tenosynovitis, and osteomyelitis. These conditions frequently require operative intervention initially and are unsuitable for outpatient management. The exception to these exclusions is the limitation of ICD9 codes 681 × and 682 × which include cellulitis and abscess in the same code and cannot be separated.

Patient data were extracted from the electronic medical record at each institution, and the combined data were managed using Microsoft Excel (Microsoft Corporation, Redmond, Washington). Demographic data included age, all ICD9/10 codes on the encounter, admission from the ED or discharge home, antibiotic administration in the ED, antibiotic prescriptions for home, and ED bedside procedural data if available. Vitals included heart rate, mean arterial pressure (MAP), and temperature. Laboratory data included lactate, glucose, sodium, creatinine, white blood cell count (WBC), c-reactive protein (CRP), and any available specimen culture data. Along with inflammatory markers, sodium can be either elevated or decreased in the setting of infection and was included [3, 4]. Laboratory data were incomplete for the cohort as not every patient who presents to the ED undergoes bloodwork. One hospital system had no digital vitals collection that could be abstracted from the EMR. Comorbidity codes were classified according to the Elixhauser Comorbidity Measure (ECM) and Charlson Comorbidity Index (CCI) using the "Comorbidity" package in R Statistical Software [5]. Both indices provide a summative score of dichotomous unweighted variables or a weighted score for each comorbidity and are validated in the Orthopedic literature in predicting complications [6, 7]. We used unweighted and weighted scores adapted by Quan et al. for each index [8].

Data collection occurred for all patient encounters in the ED, and we performed the final analysis on information obtained during the initial encounter. Home discharge from the ED without subsequent inpatient admission for that infection defined success. Subsequent return to the ED without admission was still a success. An encounter was considered a unique presentation for infection only if it was over 30 days from a previous presentation for an infection. Failure was an initial discharge from the ED with subsequent inpatient admission within 30 days of the last visit. We judged this an appropriate interval for treatment failures, as most hand infections should resolve within seven days when treated appropriately [2]. Previous studies on cellulitis have frequently used treatment lengths of up to 14 days, and many have defined treatment failure as recurrence within 30 days [9, 10]. Therefore, admission within 30 days was used as our definition of treatment failure. Each failure underwent a manual chart review to validate that admission was due to the initial infection. Our institutions do not utilize an ED observation unit; patients are admitted or discharged from the ED after triage.

Statistical analysis was conducted with JASP (Version 0.16; JASP Team, 2021) and R Statistical Software (v4.1.3; R Core Team 2021). Independent sample Welch's t test was used for continuous variables and Fisher's exact test for categorical variables. Benjamini & Hochberg correction for multiple testing was used to adjust p-values, and adjusted values are reported as q-values. The significance for all tests was set at 0.05. Multivariable logistic regression modeling was performed on variables with bivariate p-value ≤ 0.2. There was no external funding source.

## Results

**Fig. 1:**
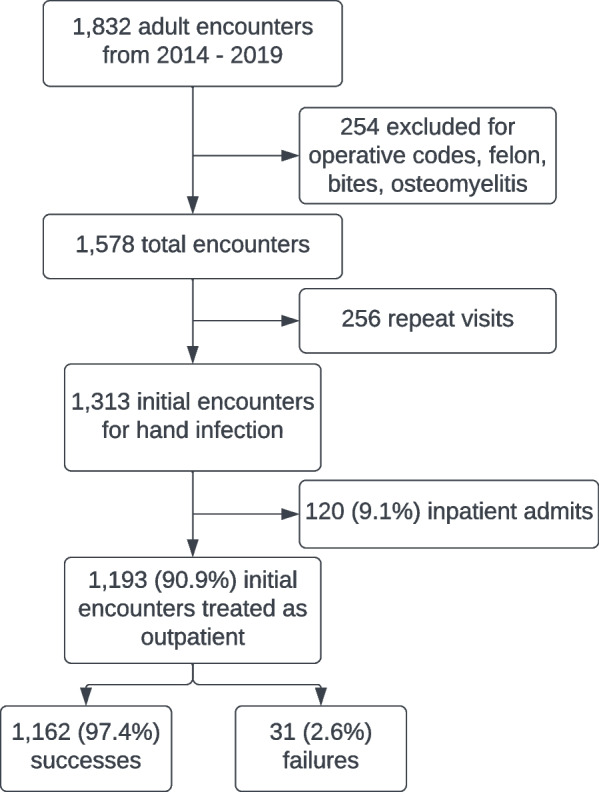
Selection of patients for outpatient treatment of hand infections

From January 1, 2014, through April 30, 2019, there were 1,832 ED encounters identified with the selected hand ICD codes, aged at least 18 years. 254 encounters also included codes for operative conditions or infection proximal to the hand and were excluded. Of the remaining 1,578 encounters, 256 were repeat visits and were excluded leaving 1,313 initial encounters (Fig. 1). 120 (9.1%) of the 1,313 were admitted without a trial of outpatient therapy and were excluded. 1,193 infections underwent attempted outpatient therapy and were included in the final analysis. Of the 1,193 outpatients there were 31 (2.6%) treatment failures and 1,162 (97.4%) successes. The ICD codes of our included population are shown in Table 1.

**Table 1:** ICD-9-CM and ICD-10 codes present in outpatient treatment groups

ICD 9/10 code	Description	Count (N = 1365)
L03011	Cellulitis of right finger	362
L03012	Cellulitis of left finger	297
6824	Cellulitis and abscess of hand, except fingers and thumb	243
L03019	Cellulitis of unspecified finger	162
68,100	Cellulitis and abscess of finger, unspecified	144
68,102	Onychia and paronychia of finger	117
6819	Cellulitis and abscess of unspecified digit	33
9141	Abrasion hand infected	3
L03021	Acute lymphangitis of right finger	2
L03022	Acute lymphangitis of left finger	1
9151	Abrasion or friction burn of finger(s), infected	1

The mean age was 47.3 ± 18.7 years overall and was not different between groups. No lab markers significantly differed between failure and success (Table 2). Length of stay in the ED was higher in the failure group (*p* = 0.003, *q* = 0.035), and both were less than 4 h on average, confirming the discharge of all patients without using an extended stay ED observation unit. On bivariate analysis, antibiotic use in the ED had higher odds of failure, which did not maintain significance after multiple testing adjustments (OR 2.31, *p* = 0.028, *q* = 0.087) (Table 3). Procedural ICD data were available for two of the hospital systems. Having a bedside irrigation & debridement procedure in the ED was not associated with lower odds of failure.

**Table 2:** Demographic and laboratory values for success and failure groups

Characteristic	Success, N = 1,162^a^	Failure, N = 31^a^	p-value^b^	q-value^*c*^
Age	47.18 (18.7)	51.55 (16.6)	0.2	0.6
LOS days	0.10 (0.1)	0.15 (0.1)	0.003	0.035
Cr mg/dL	1.02 (0.7)	1.44 (1.8)	0.4	0.6
Missing (n)	988	19		
CRP mg/dL	3.04 (4.2)	5.33 (7.4)	0.5	0.6
Missing (n)	1,082	24		
Glucose mg/dL	133.25 (78.6)	150.14 (82.3)	0.5	0.6
Missing (n)	984	17		
Lactate mmol/L	1.42 (0.6)	1.13 (0.3)	0.2	0.6
Missing (n)	1,126	27		
Na meq/L	137.57 (3.4)	136.17 (4.4)	0.3	0.6
Missing (n)	988	19		
ESR mm/Hr	25.66 (22.4)	32.33 (30.4)	0.6	0.7
Missing (n)	1,100	25		
WBC K/uL	9.42 (3.4)	9.72 (3.4)	0.8	0.8
Missing (n)	974	18		
Heart rate	84.56 (50.4)	79.44 (21.2)	0.4	0.6
Missing (n)	590	15		
Temperature	36.79 (0.3)	36.13 (2.3)	0.2	0.6
Missing (n)	492	13		

^a^Mean (SD)

^b^Welch two-sample t test

^c^Benjamini & Hochberg correction for multiple testing

**Table 3:** Procedures and antibiotic use in the success and failure groups

Characteristic	Success, N = 1,162^a^	Failure, N = 31^a^	OR (95% CI)^b^	p-value^c^	q-value^d^
Bedside procedure	197 (20%)	5 (18%)	0.86 (0.25, 2.35)	> 0.9	> 0.9
Missing (n)	186	3			
Given in ED	512 (44%)	20 (65%)	2.31 (1.04, 5.38)	0.028	0.087
Given for home	868 (75%)	25 (81%)	1.41 (0.56, 4.25)	0.5	0.7
Given for both	408 (35%)	17 (55%)	2.24 (1.03, 4.97)	0.035	0.087
None	190 (16%)	3 (9.7%)	0.55 (0.11, 1.80)	0.5	0.7

^a^n (%)

^b^OR = Odds Ratio, CI = Confidence Interval

^c^Fisher's Exact Test for Count Data

^d^Benjamini & Hochberg correction for multiple testing

CCI and ECM scores were computed for each group. In a bivariate analysis of the CCI, diabetes with and without complications and renal disease were significant though only renal disease remained so after adjustment (OR 11.1, *p* = 0.005, *q* = 0.019) (Table 4). ECM analysis found hypertension without complications, diabetes without complications, and renal failure to be significant on bivariate analysis, but none remained significant after adjustment for multiple testing (Table 5). We compared summative unweighted and weighted ECM and CCI scores between groups, and no difference was observed after adjustment (Additional File 1). Multivariable logistic regression models were built for CCI and ECM variables with bivariate p-value ≤ 0.2. The CCI model showed complicated diabetes (OR 18.29, *p* = 0.021, *q* = 0.032) and renal failure (OR 10.2, *p* < 0.001, *q* = 0.002) are associated with higher odds of failure. The ECM model demonstrated that only renal failure had increased odds of failure (OR 12.63, *p* = 0.003, *q* = 0.01) (Table 6).

**Table 4:** Charlson Comorbidity Index for success and failure groups

Charlson comorbidity	Success, N = 1,162^a^	Failure, N = 31^a^	OR (95% CI)^b^	p-value^c^	q-value^d^
Chronic pulmonary disease	35 (3.0%)	1 (3.2%)	1.07 (0.03, 6.86)	0.6	0.6
Diabetes with complication	2 (0.2%)	1 (3.2%)	19.1 (0.32, 376)	0.076	0.1
Diabetes without complication	126 (11%)	7 (23%)	2.40 (0.85, 5.88)	0.073	0.1
Renal disease	11 (0.9%)	3 (9.7%)	11.1 (1.89, 45.4)	0.005	0.019
AIDS/HIV	1 (< 0.1%)				
Cancer	9 (0.8%)				
Congestive heart failure	8 (0.7%)				
Dementia	3 (0.3%)				
Mild liver disease	7 (0.6%)				
Myocardial infarction	2 (0.2%)				
Peptic ulcer disease	1 (< 0.1%)				
Peripheral vascular disease	3 (0.3%)				
Rheumatoid disease	14 (1.2%)				

^a^n (%)

^b^OR = Odds Ratio, CI = Confidence Interval

^c^Fisher's Exact Test for Count Data

^d^Benjamini & Hochberg correction for multiple testing

**Table 5:** Elixhauser comorbidity measure for success and failure groups

Elixhauser comorbidity	Success, N = 1,162^a^	Failure, N = 31^a^	OR (95% CI)^b^	p-value^c^	q-value^d^
AIDS/HIV	1 (< 0.1%)	0 (0%)			
Cardiac arrhythmias	24 (2.1%)	0 (0%)			
Coagulopathy	1 (< 0.1%)	0 (0%)			
Congestive heart failure	8 (0.7%)	0 (0%)			
Hypothyroidism	45 (3.9%)	0 (0%)			
Liver disease	7 (0.6%)	0 (0%)			
Lymphoma	1 (< 0.1%)	0 (0%)			
Obesity	11 (0.9%)	0 (0%)			
Other neurological disorders	15 (1.3%)	0 (0%)			
Peptic ulcer disease	1 (< 0.1%)	0 (0%)			
Peripheral vascular disorders	3 (0.3%)	0 (0%)			
Psychoses	8 (0.7%)	0 (0%)			
Pulmonary circulation disorders	2 (0.2%)	0 (0%)			
Solid tumor, without metastasis	6 (0.5%)	0 (0%)			
Valvular disease	4 (0.3%)	0 (0%)			
Weight loss	1 (< 0.1%)	0 (0%)			
Alcohol use disorder	15 (1.3%)	1 (3.2%)	2.55 (0.06, 17.6)	0.3	0.5
Chronic pulmonary disease	35 (3.0%)	1 (3.2%)	1.07 (0.03, 6.86)	0.6	0.7
Depression	18 (1.5%)	1 (3.2%)	2.12 (0.05, 14.3)	0.4	0.5
Diabetes, complicated	15 (1.3%)	1 (3.2%)	2.55 (0.06, 17.6)	0.3	0.5
Drug use disorder	37 (3.2%)	1 (3.2%)	1.01 (0.02, 6.46)	> 0.9	> 0.9
Fluid and electrolyte disorders	12 (1.0%)	1 (3.2%)	3.19 (0.07, 22.9)	0.3	0.5
Hypertension, complicated	9 (0.8%)	1 (3.2%)	4.26 (0.09, 32.5)	0.2	0.5
Rheumatoid disease	15 (1.3%)	1 (3.2%)	2.55 (0.06, 17.6)	0.3	0.5
Hypertension, uncomplicated	196 (17%)	10 (32%)	2.34 (0.97, 5.30)	0.049	0.2
Renal failure	11 (0.9%)	3 (9.7%)	11.1 (1.89, 45.4)	0.005	0.052
Diabetes, uncomplicated	112 (9.6%)	7 (23%)	2.73 (0.97, 6.72)	0.028	0.2

^a^n (%)

^b^CI = Confidence Interval

^c^Fisher's Exact Test for Count Data

^d^Benjamini & Hochberg correction for multiple testing

**Table 6:** Logistic regression models for Charlson and Elixhauser comorbidities

	OR (95% CI)^a^	p-value	q-value^b^
Charlson Comorbidity Index			
Diabetes, complicated	18.29 (1.55, 216.00)	0.021	0.032
Diabetes, uncomplicated	2.06 (0.84, 5.03)	0.114	0.114
Renal Failure	10.20 (2.63, 39.51)	< .001	0.002
Elixhauser comorbidity measure			
Diabetes, uncomplicated	1.94 (0.72, 5.22)	0.187	0.249
Hypertension, complicated	0.61 (0.04, 8.83)	0.714	0.714
Hypertension, uncomplicated	1.82 (0.75, 4.40)	0.182	0.249
Renal Failure	12.63 (2.43, 65.63)	0.003	0.01

^a^OR = Odds Ratio, CI = Confidence Interval

^b^Benjamini & Hochberg correction for multiple testing

Antibiotic choice and class were compared for each group for ED use and home prescription. No significant difference between groups was found for the antibiotic type or class (Additional File 1). Culture data were available for 71 patients and demonstrated no difference between groups. However, the small sample size in the failure group limits this analysis (Table 7).

**Table 7:** Cultured species for success and failure groups

Culture	Failure	Success	*P* value^a^	*q*-value^b^
*Staphylococcus aureus*	0	17		
*Methicillin-resistant Staphylococcus aureus*	1	12		
*Streptococcus pyogenes*	0	9		
*Coagulase-negative Staphylococci*	0	6		
*Group B Streptococcus*	0	3		
*Klebsiella pneumoniae*	0	3		
*Enterobacter cloacae complex*	0	2		
*Escherichia coli*	0	2		
*Haemophilus influenzae*	0	2		
*Polymicrobial*	0	2		
*Acinetobacter lwoffi*	0	1		
*Acinetobacter radioresistens*	0	1		
*Diphtheroid*	1	1		
*Eikenella corrodens*	0	1		
*Haemophilus parainfluenzae*	0	1		
*Klebsiella oxytoca*	0	1		
*Methicillin-resistant coagulase-negative Staphylococci*	0	1		
*Prevotella intermedia*	0	1		
*Sphingomonas paucimobilis*	0	1		
*Streptococcus anginosus*	0	1		
*Streptococcus constellatus*	0	1		
Total	2	69	0.445	0.445

^a^Fisher's exact test

^b^Benjamini & Hochberg correction for multiple testing

## Discussion

For medically managed hand infections, risk factors portending outpatient failure are unclear. Our study used the validated Elixhauser and Charlson Comorbidity Indices and demonstrated that renal failure was associated with higher odds of outpatient failure in both multivariable regression models. Diabetes with complications had higher odds of failure in the CCI model. The administration of antibiotics in the ED and the need for both ED and home prescriptions were significant on bivariate analysis but not after adjustment for multiple testing. The use of antibiotics likely serves as a surrogate marker for the perceived clinical severity of an infection. We are aware of no standardized or widespread assessment tools, and previous reviews have demonstrated a lack of standardized definitions when dealing with cellulitic infections [10]. The recent creation of a Cellulitis Severity Score may serve as a more reliable decision-making tool for antibiotic administration [11]. The perceived clinical severity may be based on the visual appearance of a cellulitic infection, such as the area involved or the erythema and color. These have not, to our knowledge, been validated as a metric of severity and are not routinely or accurately reported in medical records at our institutions. Future studies are needed to compare outcomes with and without ED antibiotic administration in hand infections using such scoring systems. We demonstrated here that the administration of antibiotics in the ED for what may be deemed more severe infections did not significantly change the overall odds of failure of outpatient treatment. The increased length of stay in the ED during triage for failures could be attributed to the increased use of antibiotics. It has been previously demonstrated for soft tissue infections that receiving a first dose of antibiotics in the ED adds significant time to discharge [12].

While patients with renal and diabetic comorbidities were likelier to fail, our population's overall success rate for outpatient therapy of cellulitic hand infections was 97.4%. During the initial triage, it is imperative to distinguish which patients require emergent operative intervention. An appropriate diagnosis and surgical management of abscesses and infections involving the joint or tendon sheath cannot be neglected. Our study population was those who did not have an operative diagnosis at the presentation. These patients comprise the most common population triaged in an ED. Paronychia, and even felon, can be effectively managed as outpatients without antibiotics after appropriate I&D in the ED, and we did not demonstrate a difference for patients who required ED procedures [13].

The CCI model predicted complicated diabetes to be a risk for failure, while the ECM did not find complicated or uncomplicated diabetes to be a risk. However, these indexes use different ICD codes for diabetes with and without complications. In a cohort of diabetic and nondiabetic hand infections treated as outpatients, Qasawa et al. found a low rate of outpatient failure [14]. Their failure rate was 7% for nondiabetics and 9% for diabetics, higher than we reported here. Diabetes is often seen as a comorbidity along with renal disease. Xu et al. found it was the cause of renal failure in 88% of ESRD patients who underwent upper extremity infection surgical debridement [15]. Patients with renal disease and diabetes demand extra consideration, and prospective studies are needed to determine the safest course of treatment for this complex group. Sharma et al. showed diabetic patients were more likely to require inpatient management after I&D for upper extremity infection, demonstrating the importance of risk factor recognition during triage [16].

Kiran et al. developed an algorithmic approach to treating MRSA infections, which is generalizable to all hand infections and provides an excellent framework for triage [17]. It is beyond the scope of this article to provide recommendations on specific antibiotic therapies, and many references are available for review on drug choice depending on organism prevalence and individual hospital antibiograms [18–22]. We did not demonstrate any regiment to be more predictive of failure.

There are limitations to our study. First, our laboratory and vital data dataset was incomplete for the entire patient cohort, which may have underpowered the ability to detect a difference in these factors. Laboratory workup is not indicated in all patients on initial triage; therefore, the incompletion in collected labs represents the typical case of patients triaged in the ED.

Perhaps the most significant limitation of the study is the dependence on accurate medical diagnosis coding. The accuracy of retrospective patient identification using the discharge diagnosis relies on the ED provider and medical coders correctly identifying and coding each infection encounter. While no specific literature was available on the accuracy of extremity infection coding, multiple articles on the accuracy of ICD coding demonstrate heterogeneity in the reliability of coded data. In a recent meta-analysis, Redondo-Gonzalez et al. demonstrated that coded data could be up to 95% sensitive in identifying prosthetic surgical site infections (SSIs) while only 65% sensitive for non-prosthetic SSIs [23]. Algorithmic identification of patients with ICD9 and READ codes for infection while using oral anti-diabetic drugs was found to have 83% positive predictive value in a Medicare database and 89% PPV in the HIRD database [24]. Similarly, Lo Re et al. found an 80% PPV for identifying severe infections among patients on biologic therapy using ICD10 discharge codes [25]. The body of literature on the accuracy and PPV of diagnosis coding suggests it is helpful for retrospective studies such as ours. However, there is a need for further prospective studies to confirm retrospective findings. The strengths of our study are the multi-center nature and large sample population, as well as our multivariable statistical analysis.

## Conclusion

According to Charlson and Elixhauser Comorbidity Indices, patients with renal disease demonstrated significantly higher odds of outpatient therapy failure for cellulitic hand infections. Patients with complicated diabetes, according to Charlson but not Elixhauser Comorbidity Indices, also demonstrated increased odds of failure. These risk factors demand careful consideration for inpatient therapy. However, we found an overall success rate of 97.4%, suggesting most hand infections triaged in the ED may safely undergo outpatient treatment.

## Supplementary Information

**Additional file 1:** Additional tables representing full list of ICD codes searched, ECM and CCI weighted and unweighted scores between groups, and antibiotic type and class between groups.

## Data Availability

The datasets generated and/or analyzed during the current study are not publicly available due to patient confidentiality but are available from the corresponding author upon reasonable request.

## Abbreviations

CCI: Charlson Comorbidity Index
CRP: C-reactive protein
ECM: Elixhauser Comorbidity Measure
ED: Emergency Department
ESR: Erythrocyte sedimentation rate
ICD: International Classification of Diseases
I&D: Irrigation and Debridement
MAP: Mean Arterial Pressure
WBC: White Blood Cell
