# Supplementary material for: Renal disease and diabetes increase the risk of failed outpatient management of cellulitic hand infections: a retrospective cohort study
Source: J Orthop Surg Res. 2023 Jun 10;18:420. doi: 10.1186/s13018-023-03911-5 (PMC10257317; doi:10.1186/s13018-023-03911-5)
Supplement: Supplementary file 1 — Additional file 1: Additional tables representing full list of ICD codes searched, ECM and CCI weighted and unweighted scores between groups, and antibiotic type and class between groups. [file 13018_2023_3911_MOESM1_ESM.docx]

Supplemental Table 1 – The Included ICD-9 and ICD-10 Codes For Hand Infections

| Code | Description |
| --- | --- |
| ICD9 |  |
| 6810 | Cellulitis and abscess of finger |
| 6819 | Cellulitis and abscess of unspecified digit |
| 6824 | Cellulitis and abscess of hand, except fingers and thumb |
| 9141 | Abrasion hand infected |
| 9143 | Blister hand infected |
| 9151 | Abrasion finger infected |
| 9153 | Blister finger infected |
|  |  |
| ICD10 | |
| L0301 | Cellulitis of finger |
| L03011 | Cellulitis of right finger |
| L03012 | Cellulitis of left finger |
| L03019 | Cellulitis of unspecified finger |
| L0302 | Acute lymphangitis of finger |
| L03021 | Acute lymphangitis of right finger |
| L03022 | Acute lymphangitis of left finger |
| L03029 | Acute lymphangitis of unspecified finger |

Supplemental Table 2 – Weighted and Unweighted Elixhauser and Charlson Comorbidity Scores for Success and Failure Groups

| **Characteristic** | **Success**, N = 1,162*^1^* | **Failure**, N = 31*^1^* | **p-value***^2^* | **q-value***^3^* |
| --- | --- | --- | --- | --- |
| Elixhauser Unweighted | |  | 0.074 | 0.1 |
| 0 | 778 (67%) | 14 (45%) |  |  |
| 1 | 222 (19%) | 9 (29%) |  |  |
| 2 | 112 (9.6%) | 5 (16%) |  |  |
| 3 | 38 (3.3%) | 3 (9.7%) |  |  |
| 4 | 7 (0.6%) | 0 (0%) |  |  |
| 5 | 5 (0.4%) | 0 (0%) |  |  |
| Elixhauser Weighted | |  | 0.3 | 0.3 |
| -10 | 3 (0.3%) | 1 (3.2%) |  |  |
| -7 | 27 (2.3%) | 0 (0%) |  |  |
| -4 | 10 (0.9%) | 0 (0%) |  |  |
| -3 | 7 (0.6%) | 0 (0%) |  |  |
| -1 | 5 (0.4%) | 0 (0%) |  |  |
| 0 | 1,013 (87%) | 25 (81%) |  |  |
| 1 | 1 (<0.1%) | 0 (0%) |  |  |
| 2 | 2 (0.2%) | 0 (0%) |  |  |
| 3 | 26 (2.2%) | 1 (3.2%) |  |  |
| 4 | 6 (0.5%) | 0 (0%) |  |  |
| 5 | 33 (2.8%) | 4 (13%) |  |  |
| 6 | 7 (0.6%) | 0 (0%) |  |  |
| 7 | 2 (0.2%) | 0 (0%) |  |  |
| 8 | 3 (0.3%) | 0 (0%) |  |  |
| 9 | 3 (0.3%) | 0 (0%) |  |  |
| 10 | 4 (0.3%) | 0 (0%) |  |  |
| 11 | 5 (0.4%) | 0 (0%) |  |  |
| 12 | 3 (0.3%) | 0 (0%) |  |  |
| 16 | 1 (<0.1%) | 0 (0%) |  |  |
| 17 | 1 (<0.1%) | 0 (0%) |  |  |
| Charlson Unweighted | |  | 0.041 | 0.1 |
| 0 | 969 (83%) | 22 (71%) |  |  |
| 1 | 167 (14%) | 6 (19%) |  |  |
| 2 | 23 (2.0%) | 3 (9.7%) |  |  |
| 3 | 3 (0.3%) | 0 (0%) |  |  |
| Charlson Weighted | |  | 0.072 | 0.1 |
| 0 | 1,081 (93%) | 26 (84%) |  |  |
| 1 | 50 (4.3%) | 5 (16%) |  |  |
| 2 | 25 (2.2%) | 0 (0%) |  |  |
| 3 | 4 (0.3%) | 0 (0%) |  |  |
| 4 | 2 (0.2%) | 0 (0%) |  |  |
| *^1^* n (%) | | | | |
| *^2^* Fisher’s exact test | | | | |
| *^3^* Benjamini & Hochberg correction for multiple testing | | | | |

Supplemental Table 3 – Antibiotic Selection For ED And Home In Success And Failure Groups

| Characteristic | Success,  N = 1,162*^1^* | Failure,  N = 31*^1^* | p-value*^2^* | q-value*^3^* |
| --- | --- | --- | --- | --- |
| Ed Antibiotic Type |  |  | 0.019 | 0.078 |
| Amoxicillin/Clavulanate | 9 (1.8%) | 1 (5.0%) |  |  |
| Ampicillin/Sulbactam | 5 (1.0%) | 1 (5.0%) |  |  |
| Bacitracin | 12 (2.3%) | 0 (0%) |  |  |
| Cefazolin | 11 (2.1%) | 1 (5.0%) |  |  |
| Ceftriaxone | 57 (11%) | 6 (30%) |  |  |
| Cephalexin | 156 (30%) | 2 (10%) |  |  |
| Ciprofloxacin | 2 (0.4%) | 0 (0%) |  |  |
| Clindamycin | 66 (13%) | 1 (5.0%) |  |  |
| Daptomycin | 3 (0.6%) | 1 (5.0%) |  |  |
| Doxycycline | 28 (5.5%) | 0 (0%) |  |  |
| Ertapenem | 1 (0.2%) | 0 (0%) |  |  |
| Levofloxacin | 1 (0.2%) | 0 (0%) |  |  |
| Linezolid | 6 (1.2%) | 1 (5.0%) |  |  |
| Piperacillin/Tazobactam | 2 (0.4%) | 0 (0%) |  |  |
| Trimethoprim/Sulfamethoxazole | 123 (24%) | 4 (20%) |  |  |
| Vancomycin | 30 (5.9%) | 2 (10%) |  |  |
| (Missing) | 650 | 11 |  |  |
| Home Antibiotic Type |  |  | 0.8 | 0.8 |
| Amoxicillin/Clavulanate | 27 (3.1%) | 2 (8.0%) |  |  |
| Amoxicillin/Clavulanate,   Trimethoprim/Sulfamethoxazole | 2 (0.2%) | 0 (0%) |  |  |
| Bacitracin | 4 (0.5%) | 0 (0%) |  |  |
| Cefdinir | 1 (0.1%) | 0 (0%) |  |  |
| Cefuroxime | 1 (0.1%) | 0 (0%) |  |  |
| Cephalexin | 381 (44%) | 9 (36%) |  |  |
| Cephalexin, Doxycycline | 1 (0.1%) | 0 (0%) |  |  |
| Cephalexin, Trimethoprim/Sulfamethoxazole | 26 (3.0%) | 0 (0%) |  |  |
| Ciprofloxacin | 2 (0.2%) | 0 (0%) |  |  |
| Clindamycin | 28 (3.2%) | 1 (4.0%) |  |  |
| Clindamycin | 85 (9.8%) | 3 (12%) |  |  |
| Clindamycin, Doxycycline | 1 (0.1%) | 0 (0%) |  |  |
| Doxycycline | 53 (6.1%) | 2 (8.0%) |  |  |
| Doxycycline, Trimethoprim/Sulfamethoxazole | 1 (0.1%) | 0 (0%) |  |  |
| Levofloxacin | 1 (0.1%) | 0 (0%) |  |  |
| Linezolid | 3 (0.3%) | 0 (0%) |  |  |
| Mupirocin | 5 (0.6%) | 0 (0%) |  |  |
| Trimethoprim/Sulfamethoxazole | 246 (28%) | 8 (32%) |  |  |
| (Missing) | 294 | 6 |  |  |
| Ed Antibiotic Class |  |  | 0.2 | 0.3 |
| B-Lactam/B-Lactamase Inhibitor Combination | 16 (3.1%) | 2 (10%) |  |  |
| Carbapenems | 1 (0.2%) | 0 (0%) |  |  |
| Cephalosporins | 224 (44%) | 9 (45%) |  |  |
| Fluoroquinolones | 3 (0.6%) | 0 (0%) |  |  |
| Glycopeptides | 30 (5.9%) | 2 (10%) |  |  |
| Lincosamides | 66 (13%) | 1 (5.0%) |  |  |
| Lipopeptides | 3 (0.6%) | 1 (5.0%) |  |  |
| Oxazolidinones | 6 (1.2%) | 1 (5.0%) |  |  |
| Polypeptide | 12 (2.3%) | 0 (0%) |  |  |
| Tetracyclines | 28 (5.5%) | 0 (0%) |  |  |
| Trimethoprim/Sulfamethoxazole | 123 (24%) | 4 (20%) |  |  |
| Home Antibiotic Class |  |  | 0.8 | 0.8 |
| B-Lactam/B-Lactamase Inhibitor Combination | 27 (3.1%) | 2 (8.0%) |  |  |
| B-Lactam/B-Lactamase Inhibitor Combination,   Trimethoprim/Sulfamethoxazole | 2 (0.2%) | 0 (0%) |  |  |
| Carboxylic Acid | 5 (0.6%) | 0 (0%) |  |  |
| Cephalosporins | 383 (44%) | 9 (36%) |  |  |
| Cephalosporins, Tetracyclines | 1 (0.1%) | 0 (0%) |  |  |
| Cephalosporins, Trimethoprim/Sulfamethoxazole | 26 (3.0%) | 0 (0%) |  |  |
| Fluoroquinolones | 3 (0.3%) | 0 (0%) |  |  |
| Lincosamides | 113 (13%) | 4 (16%) |  |  |
| Lincosamides, Tetracyclines | 1 (0.1%) | 0 (0%) |  |  |
| Oxazolidinones | 3 (0.3%) | 0 (0%) |  |  |
| Polypeptide | 4 (0.5%) | 0 (0%) |  |  |
| Tetracyclines | 53 (6.1%) | 2 (8.0%) |  |  |
| Tetracyclines, Trimethoprim/Sulfamethoxazole | 1 (0.1%) | 0 (0%) |  |  |
| Trimethoprim/Sulfamethoxazole | 246 (28%) | 8 (32%) |  |  |
| *^1^* N (%) | | | |  |
| *^2^* Fisher’s Exact Test | | | |  |
| *^3^* Benjamini & Hochberg Correction For Multiple Testing | | | |  |
